# Assessment of pregnancy dietary intake and association with maternal and neonatal outcomes

**DOI:** 10.1038/s41390-021-01665-6

**Published:** 2021-08-03

**Authors:** Jole Costanza, Margherita Camanni, Maria Maddalena Ferrari, Valentina De Cosmi, Silvia Tabano, Laura Fontana, Tatjana Radaelli, Giulia Privitera, Daniela Alberico, Patrizia Colapietro, Silvia Motta, Silvia Sirchia, Tamara Stampalija, Chiara Tabasso, Paola Roggero, Fabio Parazzini, Fabio Mosca, Enrico Ferrazzi, Silvano Bosari, Monica Miozzo, Carlo Agostoni

**Affiliations:** 1 Research Laboratories Coordination Unit, Fondazione IRCCS Ca’ Granda Ospedale Maggiore Policlinico, Milan, Italy; 2 Unit of Obstetric, Department of Woman, Child and Neonate “L. Mangiagalli”, Fondazione IRCCS Ca’ Granda Ospedale Maggiore Policlinico, Milan, Italy; 3 Pediatric Intermediate Care Unit, Fondazione IRCCS Ca’ Granda Ospedale Maggiore Policlinico, Milan, Italy; 4 Department of Clinical Sciences and Community Health, Università degli Studi di Milano, Milan, Italy; 5 Laboratory of Medical Genetics, Fondazione IRCCS Ca Granda Ospedale Maggiore Policlinico, Milan, Italy; 6 Department of Pathophysiology & Transplantation, Università degli Studi di Milano, Milan, Italy; 7 Medical Genetics, Department of Health Sciences, Università degli Studi di Milano, Milan, Italy; 8 Unit of Fetal Medicine and Prenatal Diagnosis, Institute for Maternal and Child Health IRCCS Burlo Garofolo, Trieste, Italy; 9 Department of Medical, Surgical and Health Science, Università diTrieste, Trieste, Italy; 10 Neonatal Intensive Care Unit, Fondazione IRCCS Cà Granda Ospedale Maggiore Policlinico, Milan, Italy; 11 Scientific Direction, Fondazione IRCCS Ca’ Granda Ospedale Maggiore Policlinico, Milan, Italy

## Abstract

**Background:**

Maternal dietary habits are contributors of maternal and fetal health; however, available data are heterogeneous and not conclusive.

**Methods:**

Nutrient intake during pregnancy was assessed in 503 women with uncomplicated pregnancies, using the validated Food Frequency Questionnaire developed by the European Prospective Investigation into Cancer and Nutrition (EPIC-FFQ).

**Results:**

In all, 68% of women had a normal body mass index at the beginning of pregnancy, and 83% of newborns had an appropriate weight for gestational age. Maternal pre-pregnancy body mass index (BMI), gestational weight gain (GWG), and placental weight were independently correlated with birth weight. GWG was not related to the pre-pregnancy BMI. EPIC-FFQ evaluation showed that 30% of women adhered to the European Food Safety Authority (EFSA) ranges for macronutrient intake. In most pregnant women (98.1%), consumption of water was below recommendations. Comparing women with intakes within EFSA ranges for macronutrients with those who did not, no differences were found in BMI, GWG, and neonatal or placental weight. Neither maternal nor neonatal parameters were associated with the maternal dietary profiles.

**Conclusions:**

In our population, maternal pre-pregnancy BMI, GWG, and placental weight are determinants of birth weight percentile, while no association was found with maternal nutrition. Future studies should explore associations through all infancy.

**Impact:**

- Maternal anthropometrics and nutrition status may affect offspring birth weight.
- In 503 healthy women, maternal pre-pregnancy body mass index (BMI), gestational weight gain (GWG), and placental weight were independently correlated to neonatal birth weight. GWG was not related to the pre-pregnancy BMI. In all, 30% of women respected the EFSA ranges for macronutrients. Neither maternal nor neonatal parameters were associated with maternal dietary profiles considered in this study.
- Maternal pre-pregnancy BMI, GWG, and placental weight are determinants of neonatal birth weight percentile, while a connection with maternal nutrition profiles was not found.

## Introduction

Maternal dietary habits are lifestyle-related contributors of maternal and fetal health, impacting pre-pregnancy body mass index (BMI), maternal gestational weight gain (GWG), and fetal growth.^1^ Furthermore, according to the hypothesis of Developmental Origins of Health and Disease, nutritional exposure and the subsequent metabolic programming that occurs in utero may also influence offspring physiology and metabolism later in life.^2^ Both undernutrition and overnutrition during pregnancy have been associated with clinical complications including hypertensive disorders of pregnancy and gestational diabetes, which can lead to adverse neonatal and infant conditions, such as abnormal birth weight, anatomic and functional neurodevelopmental conditions, and adulthood cardiovascular disorders.^3–6^ Birth weight could be a predictor of offspring health and placental weight identified as a determinant of intrauterine growth. In turn, placental weight is related to maternal conditions.^7,8^

Maternal dietary exposure can be monitored through GWG and pre-pregnancy BMI. A number of negative pregnancy outcomes have been linked with high and low GWG; however, limited evidence is available on the impact of optimal GWG on pregnancy outcomes.^9,10^ According to the Institute of Medicine (IOM) recommendations,^11^ GWG should be progressive and proportional to pre-pregnancy BMI. Controlling dietary intake during pregnancy allows sufficient provision of energy to the growing fetus, while keeping GWG within recommended ranges. The main energy source during gestation should be carbohydrates, which should account for 45–60% of total daily energy intake (EI), with sugar consumption within 10% of total carbohydrate intake. Fat should comprise around 30% EI, with protein contributing the remaining portion of energy.^12^ Overall EI should be adjusted for age and level of physical activity.^13^

Despite the recognized role of nutrition in pregnancy on maternal and offspring outcomes, available data are heterogeneous, mainly because of differences in study designs, dietary intake measurements, environmental confounders, and the large variability of maternal dietary habits.

Aims of the present study were to explore the macronutrient and daily EIs of European women, compare the results with the European Food Safety Authority (EFSA) recommendations and investigate the relationship between maternal nutritional status and neonatal anthropometric outcomes.

To this aim, we have conducted a survey on a cohort of European pregnant women at term with cultural and lifestyle habits consistent with the Mediterranean diet. Dietary intake was evaluated using a Food Frequency Questionnaire, developed by the European Prospective Investigation into Cancer and Nutrition questionnaire (EPIC-FFQ).^14^

## Materials and methods

Pregnant women were enrolled at the Obstetric Unit “L. Mangiagalli” at Fondazione IRCCS Ca’ Granda Ospedale Maggiore Policlinico, from September 2016, up to March 2019. Cases were selected according to the following inclusion criteria: (1) Caucasian European ancestry, (2) singleton spontaneous pregnancy delivered at ≥37 weeks of gestation, and (3) absence of fetal abnormalities. We excluded women affected by chronic diseases and/or gestational complications, such as gestational diabetes, hypertensive disorders, and/or fetal growth restrictions. The study protocol was approved by Fondazione IRCCS Ca’ Granda Ospedale Maggiore Policlinico Milano Area B Ethical Committee (reference ID number 2487-588ter (28.04.2015), and written informed consent was obtained from each woman. The enrolled women for this study belong to a more comprehensive project aimed at creating the first Italian biobank of maternal and fetal biological material from >2000 healthy pregnancies. Within the project, we will explore the Barker’s hypothesis by investigating the maternal nutrition, the fetal–placental epigenetic profile, and transcriptome patterns related to birth weight and maternal weight gain.

Herein we investigated the nutrition habits during pregnancy in a large cohort of European women. The study was designed in collaboration with clinicians, midwives, nutrition experts, and geneticists to find evidence to respond to anxiety in pregnant women about the possible consequences of their diet on newborn weight, their own health, and that of their babies.

### Data collection

Participants were enrolled at the time of hospitalization for delivery and included both cesarean section and vaginal deliveries. Maternal data, comprising anthropometric parameters (height and weight before and at the end of pregnancy), obstetric history, and clinical characteristics of the pregnancies, were obtained from medical records. Maternal nutritional habits were recorded through the EPIC-FFQ questionnaire. At the time of delivery, mode of delivery, gestational age, neonatal weight, and placental weight were recorded.

### Dietary assessment

To evaluate nutritional habits, a printed copy of the FFQ developed by the EPIC study (EPIC-FFQ) was given to participants.^14^ EPIC is a multicentric prospective cohort study investigating the relationship between diet, cancer, and other chronic diseases in over half a million participants across different European countries.^15,16^ The EPIC study was conceived by the International Agency for Research on Cancer, part of the World Health Organization, and was funded by the “Europe Against Cancer” program of the European Commission and other non-profit institutions. The questionnaire is composed of 260 multiple-choice questions supported by pictures.^14^ The EPIC-FFQ was not specifically designed to assess nutrition and/or dietary habits in pregnancy, but it has already been used by Flynn et al. in an adapted version for the UK population to assess dietary pattern in obese pregnant women.^17,18^ Each survey was processed through the licensed software EPIC (patented by Fondazione IRCCS Istituto Nazionale dei Tumori), allowing the conversion of nutritional habits into nutrient quantities per day (expressed in grams). The women filled in the EPIC-FFQ during their hospitalization for delivery. They were asked to report the nutritional habits from the first trimester up to delivery, indicating possible changes compared to their pre-pregnancy habits. The questionnaires were designed to protect respondent anonymity and to improve the reliability and accuracy of feedback, as well as to increase response rates. Macronutrient energy ratios were calculated using a formula that multiplies fat/protein/carbohydrate quantity (expressed in grams) by a standard coefficient for each macronutrient (kcal/g = 9 for fat, 4 for protein, and 3.75 for carbohydrate), according to Atwater.^19^ The results were adjusted for total daily calories. As a result, overall EI and the percentage of calories derived from carbohydrate, fat, and protein were obtained.

### Data analysis

Categorical or ordinal variables are presented as frequency (%) and continuous variables as means (standard deviation) if normally distributed, and medians (interquartile range) if not. Differences between groups were evaluated with *t* test for normally distributed variables. A one-way analysis of variance was used to evaluate differences between three or more independent groups. Correlation between birth weight and placental weight was tested with Pearson’s correlation coefficient (*r*). *K*-means clustering was performed to define dietary profiles according to macronutrient ratios. Statistical analysis and graph generation was performed in R^20^ with *p* < 0.05 considered statistically significant.

Initially, the dataset was composed by >800 women who adhered to this project; however, in order to have a homogeneous and eligible dataset, we further filtered out the population reaching the final number of 503 pregnancies. Data quality controls were indeed carried out to exclude biased entries (e.g., randomly drafted), uncompleted questionnaires (>30/260 blank answers), and those showing unlikely daily caloric intakes (<1000 kcal/day or >3500 kcal/day).

Infant growth charts developed by Bertino et al. (INeS)^21^ were used for birth weight classification. Three newborn groups were identified based on weight percentile considering weeks of gestation: (1) SGA (small for gestational age), ≤10th percentile; (2) AGA (appropriate for gestational age), >10th and <90th percentile; (3) LGA (large for gestational age), ≥90th percentile.^22,23^

Maternal weights were stratified on the basis of GWG and pre-pregnancy BMI, with women classified as underweight (BMI < 18.5 kg/m^2^), normal weight (BMI ≥ 18.5 and <25 kg/m^2^), overweight (BMI ≥ 25 and <30 kg/m^2^), and obese (BMI ≥ 30 kg/m^2^).^11^ According to IOM recommended ranges, GWG is progressive and proportional to pre-pregnancy BMI: in underweight women the recommended GWG range is 12.5–18 kg, in normal weight women 11.5–16 kg, in overweight women 7–11.5 kg, and in obese women 5–9 kg.^11^

Clinical information about each pregnancy was entered into a comprehensive database and a unique identification code was assigned to ensure privacy.

## Results

On the whole, the final dataset consists of 503 women, 474 Italian and 29 from other European countries. Clinical data are reported in Table 1.

**Table 1:** Clinical data of the study population.

	Mean ± SD	
Age (years)	35.2 ± 4.4	
Mother’s weight at delivery (kg)	69.7 ± 9.9	
Mother’s BMI at delivery (kg/m^2^)	25.9 ± 4.1	
Pre-pregnancy BMI (kg/m^2^) (*n* = 465)	***n*** **(%)**	
<18.5	55 (11.8)	
18.5–24.9	341 (73.3)	
25–30	56 (12)	
>30	12 (2.6)	
Mean ± SD	21.8 ± 3.4	
	**Primiparous**	**Multiparous**
	***n*** **(%)**	
Parity	224 (45)	2 children: 226 (45) ≥3 children: 48 (10)
	**Mean** ± **SD**	
Pre-pregnancy weight (kg)	58.5 ± 8.5 kg	60.0 ± 9.9
Pre-pregnancy BMI (kg/m^2^)	21.6 ± 3.4	21.9 ± 3.3
Gestational weight gain (kg)	10.03 ± 3.4	10.76 ± 3.3
Male placental weight (g)	585.56 ± 125	609.66 ± 114
Female placental weight (g)	572.05 ± 104	606 ± 140
Male newborn weight (g)	3351.20 ± 415	3444.83 ± 430
Female newborn weight (g)	3242.26 ± 384	3241.45 ± 407
Gestational age (weeks)	39.37 ± 1.2	39.06 ± 0.8
Mode of delivery		
Cesarean section	110 (49.2)	187 (68.2)
Vaginal	114 (50.8)	87 (31.8)

The cohort was mainly composed of women in the normal range for prenatal BMI (73.3%) and AGA newborns were delivered in 83% of cases.

Gestational age, mode of delivery, and neonatal weight were similar considering primiparous vs. multiparous women.

### Clinical outcomes

**Fig. 1:**
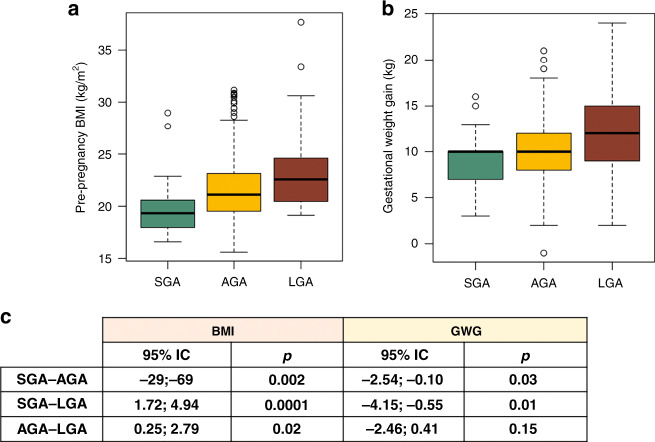
Maternal anthropometric parameters distributions based on birth weight percentile. Pre-pregnancy maternal BMI (**a**) and gestational weight gain (GWG) (**b**) boxplot distributions in small for gestational age (SGA), appropriate for gestational age (AGA), and large for gestational age (LGA) neonatal groups. **c** Pair-wise *t* test results analyzing differences in pre-pregnancy BMI and GWG between SGA, AGA, and LGA birth weight categories.

The relationships between maternal anthropometric parameters (pre-pregnancy BMI and GWG) and primary neonatal outcomes (newborn birth weight percentile and placental weight) were evaluated at the time of delivery.^24,25^ Neonatal birth weight and placental weight showed a significant positive correlation (*r* = 0.54, *r*^2^ = 0.3, *p* < 0.05; Fig. S1). When stratified by neonatal birth weight percentile groups (i.e., SGA, AGA, and LGA), there were significant differences in maternal pre-pregnancy BMI between groups (Fig. 1a). There were also significant differences in maternal GWG between SGA newborns and the other newborn classes (Fig. 1). Overall, birth weight percentile increases in parallel with GWG and pre-pregnancy BMI.

When categorized by maternal BMI, there was a higher proportion of SGA offspring from mothers with a low maternal BMI compared to other BMI categories (18% SGA in underweight women vs. 5 and 2.6% in normal weight and overweight women, respectively). There were no SGA infants born to obese mothers, who gave birth to a larger proportion of LGA infants (25%) than mothers with a lower BMI (Fig. S2).

Analysis of relative risk (RR) showed that women with a normal BMI had a lower risk of delivering SGA and LGA offspring than women in lower and higher BMI categories, respectively. The RR of a SGA birth was significant (RR = 0.27, 95% confidence interval (CI) 0.13–0.57) in normal weight compared with underweight women and the RR of a LGA newborn was not significant (RR = 0.7, 95% CI 0.34–1.38) in normal weight compared with overweight/obese women.

Additional analyses were performed to evaluate the relationships among placental weight, pre-pregnancy BMI, and GWG, revealing that placental weight correlated to pre-pregnancy BMI and GWG (Fig. S3).

Although both maternal BMI and GWG differed according to newborn weight percentile categories, maternal GWG was not different between pre-pregnancy BMI categories (*p* = 0.44; Fig. S4).

To evaluate maternal anthropometric and GWG, the study population were compared with the IOM guidelines.^11^ This comparison showed that 78% of underweight and 64% of normal weight women gained insufficient weight during pregnancy (minimum recommended thresholds 11.5 and 12.5 kg, respectively). By contrast, among obese women 58% gained more weight than the maximum recommended threshold and 25% had appropriate GWG. Most overweight women showed GWG within the recommended range (43%), while mothers with low and high GWG were equally distributed outside the lower and upper limits.

### Nutritional data

#### Daily caloric and macronutrient intake

Maternal energy requirements may vary depending on several factors, including the trimester of gestation and the level of physical activity. Since information about the physical activity levels were not available, previously reported^26^ intakes based on moderate physical activity were used as reference range (between 1800 and 2400 kcal/day). For nutrition evaluation, EI, macronutrients, fiber, and water were considered.

The mean EI in our cohort was 2108.4 ± 519.7 kcal/day. When the daily caloric intake was compared with the SIGO guidelines,^26^ 27.2% of women consumed more calories than the recommended range, 29.8% consumed less, and 43% were within the recommended range (Table 2).

**Table 2:** Recommended ranges of macronutrient and energy intake according to EFSA for fats, carbohydrates, fiber, and water.

	Recommended values	Women (%)		
	**SIGO**_**L**_**–SIGO**_**U**_	**<SIGO**_**L**_	**≥SIGO**_**L**_ **and ≤SIGO**_**U**_	**>SIGO**_**U**_
Energy	1800–2400 kcal/day	150 (29.8)	216 (43)	137 (27.2)
	**EFSA**_**L**_**–EFSA**_**U**_	**<EFSA**_**L**_	**≥EFSA**_**L**_ **and ≤EFSA**_**U**_	**>EFSA**_**U**_
Protein	12–20% EI	42 (8.3)	450 (89.5)	11 (2.2)
Fat	20–35% EI	0 (0)	185 (36.7)	318 (63.2)
Carbohydrates	45–60% EI	159 (31.6)	334 (66.4)	10 (1.9)
	**EFSA**	**<EFSA**	**>EFSA**	
Fiber	25 g	320 (63.6)	183 (36.4)	
Water	2300 mL	493 (98.1)	10 (1.9)	

Protein range is obtained by a massive EFSA European survey.^12^ For energy, the SIGO recommended range was used. Table reports the distribution of the study population in these intervals. EFSA_L_: minimum value of the interval referred to EFSA ranges; EFSA_U_: maximum value of the interval referred to EFSA ranges.^12^ SIGO_L_: minimum value of the interval referred to SIGO guidelines; SIGO_U_: maximum value of the interval referred to SIGO guidelines.^24^

Analysis of nutritional data showed that 89.5% of women respected EFSA protein range derived from a massive European survey,^27^ and only 12 women (8.3%) consumed less protein (Table 2) while the vegetable to animal protein ratio was 1:2. Around 63% of women exceeded the EFSA range for fat intake, with a vegetable to animal fat ratio of 1:1. Finally, the mean carbohydrate intake of pregnant women was within the recommended range, with 334 women (66.4%) consuming the recommended daily levels of carbohydrate.

The average intake of dietary fiber in our cohort was slightly lower than EFSA recommendation, at 23 ± 7.6 g consumed vs. 25 g recommended (Table 2). Additionally, the average consumption of water was 1152 ± 391 mL/day, about half of the recommended amounts (2300 mL/day)^28^ (Fig. S5).

To investigate associations between maternal diet and neonatal outcomes, women were divided into two groups: (1) those following EFSA recommendations for all macronutrients and (2) those who fell outside EFSA guidelines for all three macronutrients (fat, protein, and carbohydrate), to maximize possible differences at the two extremes, even considering the possible unbalance of numbers. These subsets included 151 (30%) and 11 women (2%), respectively (Table S1).

No differences were found between the two groups in terms of newborn and placental weight at birth, pre-pregnancy BMI, and GWG. However, a mild difference in birth weight was found, since women within EFSA references had children with a mean birth weight of 3355 g, while women out of EFSA references for all the three macronutrients had children with a mean birth weight of 3053 g, but the variability in this smaller group should be also accounted for.

We also evaluated birth weight values in two groups, based on “lower” vs. “higher” intakes of fat as for the cutoff of 35% indicated by EFSA recommendations and no difference has been found (*p* = 0.37, *t* test).

**Fig. 2:**
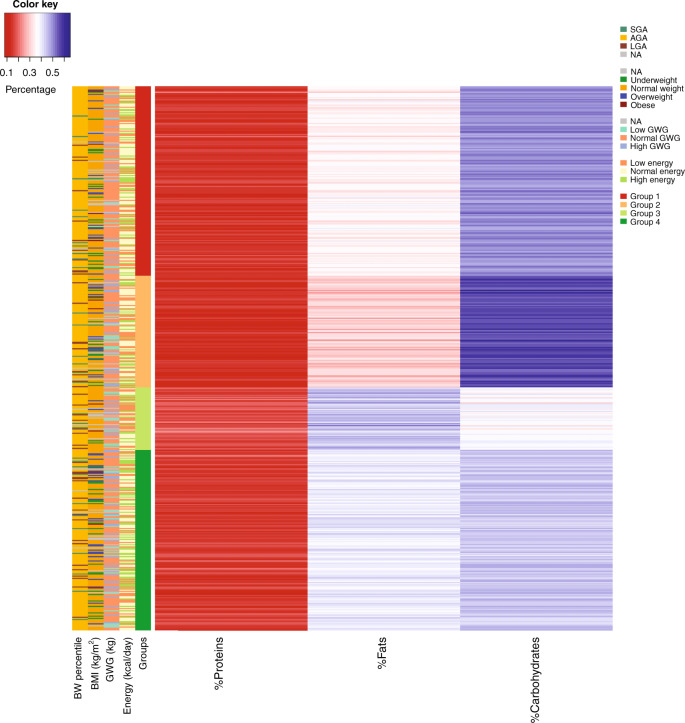
*K*-means clustering (*k* = 4) of four different dietary profiles based on macronutrient intake ratios. In red (group 1) high carbohydrate (45–55%) and normal fat (30–40%); in orange (group 2) very high carbohydrate (55–65%) and low fat (22–35%); in light green (group 3) low carbohydrate (30–40%) and very high fat (40–53%); in dark green (group 4) high carbohydrate (40–48%) and high fat (35–45%). Neonatal and maternal parameters are reported on lateral bar: birth weight percentile (BW percentile), maternal pre-pregnancy BMI, gestational weight gain (GWG), energy intake. *K*-means groups are also reported. Top-right legend reports neonatal, maternal, and nutritional categories: for birth weight percentile, infants are categorized into large for gestational age (LGA), appropriate for gestational age (AGA), small for gestational age (SGA); for maternal pre-pregnancy BMI, women are divided into underweight (BMI < 18.5 kg/m^2^), normal weight (18.5 ≤ BMI < 25 kg/m^2^), overweight (25 ≤ BMI < 30 kg/m^2^), and obese (BMI ≥ 30 kg/m^2^); for gestational weight gain (GWG), women are divided into low GWG (GWG < 7 kg), high GWG (GWG > 13 kg), and normal GWG (7 ≤ GWG ≤ 13 kg); energy intakes are divided into three categories: low energy (energy < 1200 kcal/day), normal energy (1200 ≤ energy < 2500 kcal/day), and high energy (energy > 2500 kcal/day).

Finally, *k*-means clustering was carried out, which defined four dietary profiles according to macronutrient ratios (*k* = 4) expressed in percentage according to Atwater formula, as reported in the “Materials and methods” section.^19^ The groups were mainly distinguished by fat and carbohydrate intakes. Group 1 (depicted in red in Fig. 2) included 175 women who had a high intake of carbohydrate (45–55% EI) and a normal contribution of fat (30–40% EI). Group 2 (orange) included 103 women who showed a high level of carbohydrate intake (55–65% EI) with a lower level of fat intake (22–35% EI). In addition, 58 mothers in Group 3 (light green) had a percentage of carbohydrate lower than recommended (30–40% EI) and a higher portion of fat (40–53% EI). Finally, 167 women in Group 4 (dark green) consumed a higher proportion of carbohydrate (40–48% EI) and fat (35–45% EI) than recommended. All groups fall within EFSA range for proteins, but Group 2 in particular is characterized by a relatively low protein intake (13.6% ± 1.8% EI), while in Group 3 the contribution of protein was higher (17.3% ± 2.5 EI). Neither maternal nor neonatal outcomes (pre-pregnancy BMI, GWG, newborn weight, placental weight) showed associations with these dietary profiles.

## Discussion

This study aimed to characterize dietary habits in healthy pregnant women and investigate how these related to maternal anthropometric parameters and neonatal outcomes (represented by neonatal and placental weight at birth). Neonatal outcomes have been suggested as proxy of future health status at population levels.

Neonatal and placental weight were positively correlated, with a direct association with maternal pre-pregnancy BMI and GWG, respectively. The prevalence of SGA neonates was higher in the subgroup of underweight mothers, while the prevalence of LGA neonates was higher in obese mothers. However, in the obese group, GWG was not higher than the other pre-pregnancy BMI categories. In agreement with other studies,^29–31^ our data show that excessive maternal GWG resulted in a greater proportion of LGA offspring than mothers with a lower GWG. However, in contrast to previous studies,^32–34^ we found that the relationship between GWG and offspring weight was independent of pre-pregnancy BMI.

A recent meta-analysis of the association of GWG with maternal and infant outcomes analyzed data of >1 million pregnant women and showed that 47% of women have greater GWG and 23% lower GWG, than IOM recommendations.^35^ Likewise, in our study, women did not lie within the IOM ranges for GWG. The majority of underweight and normal weight women did not reach the minimum GWG recommended (78 and 64%, respectively), while >50% obese women gained more weight than the maximum recommended GWG. Additionally, our results showed no differences in GWG and EIs between women stratified into four groups according to pre-pregnancy BMI.

The daily caloric and macronutrient intake of mothers have been investigated through the EPIC questionnaire. The repartition between macronutrients emphasized the heterogeneous dietary intakes in the sampled population. In all, 30% of the sampled population reported dietary intakes in line with all EFSA recommendations. During pregnancy, requirements of water and fiber increase, due to increased uterus weight and reduced bowel motility resulting from higher levels of progesterone.^36^ However, our data on water and fiber consumption showed that women did not reach the minimum recommended intake for either dietary component. Our results fit with the macronutrient distributions obtained from the EPIC-FFQ in another recent study even though involving a different larger Italian sample.^37^

We have also observed that maternal and neonatal outcomes (pre-pregnancy BMI, GWG, newborn weight, placental weight) were not different when mothers with different dietary profiles were compared. Since follow-up data were not available, our observations are limited to the parameters at birth. Therefore, we cannot exclude the possibility that maternal dietary habits before and during pregnancy might impact on later postnatal outcomes, such as growth and/or developmental achievements.

Our study has both strengths and limitations. A relatively large, homogeneous sample was used from a single institution and a validated FFQ was used to assess dietary habits. Although the FFQ is designed to determine eating habits over the last year, participants generally “telescope” their report backward so that their dietary information mostly reflects recent patterns of intake.^38^

Possibly, maternal weight should be put under control before, rather than during pregnancy, since optimal GWG ranges may have limited predictive value.^11^ This is also confirmed by our results suggesting that maintaining an adequate and controlled weight may represent a benefit for either maternal health or neonatal outcomes (considering birth weight and placenta).

Failure to meet recommendations for energy, protein, and fat found in the present study are in accordance with results reported for macronutrients in a cohort of 200 pregnant women by Diemert et al.^39^ and by a systematic review and meta-analysis of data from developed countries.^40^ Within this context, recommendations often focus on GWG, rather than on promoting a healthy diet as starting point during pregnancy and before conception. Despite a lack of maternal adherence to recommendations, neonatal anthropometric outcomes were within normal ranges, which may suggest compensatory fetal growth mechanisms, partly at least genetically driven, in face of maternal nutritional inadequacy. We may also speculate that the present dietary recommendation may not be relevant to the healthy local diet. The opportunity of longer-term follow-ups should be once more recommended to account for epigenetic changes and/or mechanisms with later phenotypic expression levels.^41,42^

Finally, our data cannot be directly compared with data from developing and resource-poor countries, where baseline nutritional intakes are different. For example, Pathirathna et al. studied 141 healthy pregnant women in Sri Lanka, whose dietary habits were measured by a FFQ and the results suggest that women with a total EI below recommendations delivered neonates with significantly lower mean birth weight than women who were above recommendations.^43^

In conclusion, we found that maternal pre-pregnancy BMI, GWG, and placental weight were positively correlated with neonatal birth weight. Few women had a GWG within the recommended ranges and, when looking at their nutritional habits, even fewer followed Institutional recommended intakes for energy, macronutrients, fiber, and water. Neither maternal nor neonatal outcomes were associated with the dietary profiles considered in this study. These findings suggest that nutritional counseling should be strongly implemented in pre-conceptional and obstetric clinic. As regards the apparent non-influence of inappropriate diet on newborn weight, we speculate that a longitudinal follow-up of the newborns of this cohort into their infancy could reveal a potential metabolic effect of the intrauterine environment independently from simple weight at birth—a working hypothesis requiring long-term observations in large populations from different settings.

## Supplementary information

Supplementary Material
