# Supplementary material for: Assessment of pregnancy dietary intake and association with maternal and neonatal outcomes
Source: Pediatr Res. 2021 Aug 3;91(7):1890–6. doi: 10.1038/s41390-021-01665-6 (PMC9270222; doi:10.1038/s41390-021-01665-6)
Supplement: Supplementary file 1 — Supplementary Material [file 41390_2021_1665_MOESM1_ESM.pdf]

Assessment of pregnancy dietary intake and association with maternal and neonatal outcomes - Supplementary Material

Figure S1: Placental weight and birthweight relationship.

Figure S2: Infants birthweight distribution in mothers divided by BMI.

Figure S3: Placental weight and maternal GWG/BMI relationships.

Figure S4: Gestational weight gain distributions in mothers divided by BMI.

Figure S5: Distribution of daily water (A) and fiber (B) intake in 503 women.

Table S1. Nutrition and maternal/neonatal anthropometric parameters analysis. Maternal parameters and neonatal birthweight in women following EFSA recommendations for all macronutrients (n = 151/503) and those that fell outside EFSA guidelines for fats, proteins and carbohydrates (n = 11/503).

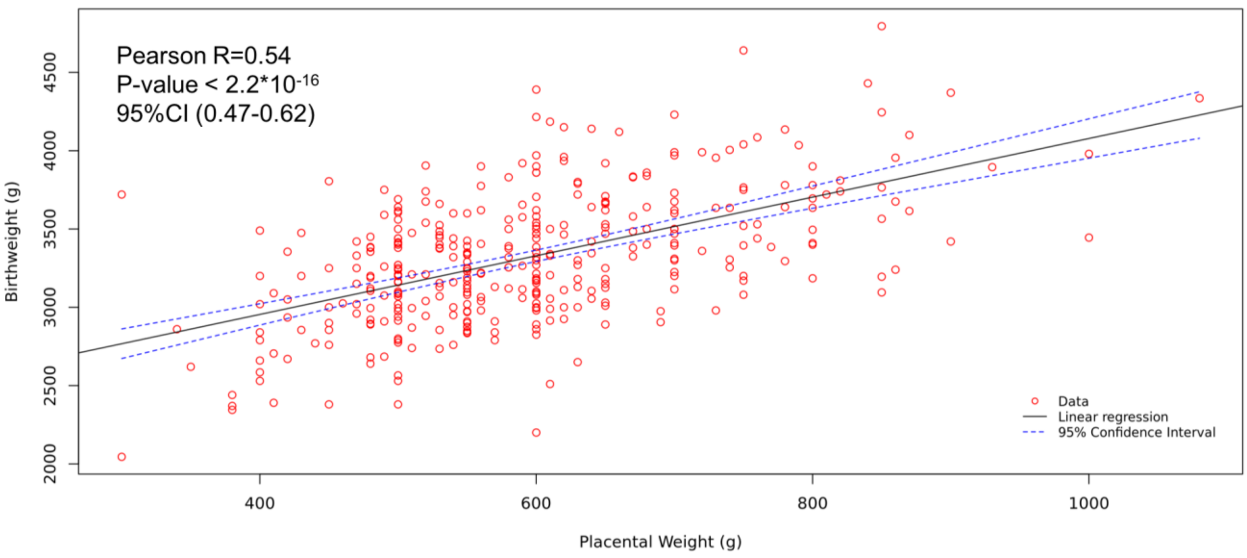

Figure S1: **Placental weight and birthweight relationship.** Scatter plot of placental weight (X-axis) and birthweight (Y- axis), measured in grams, obtained from our cohort of 503 pregnancies.

A

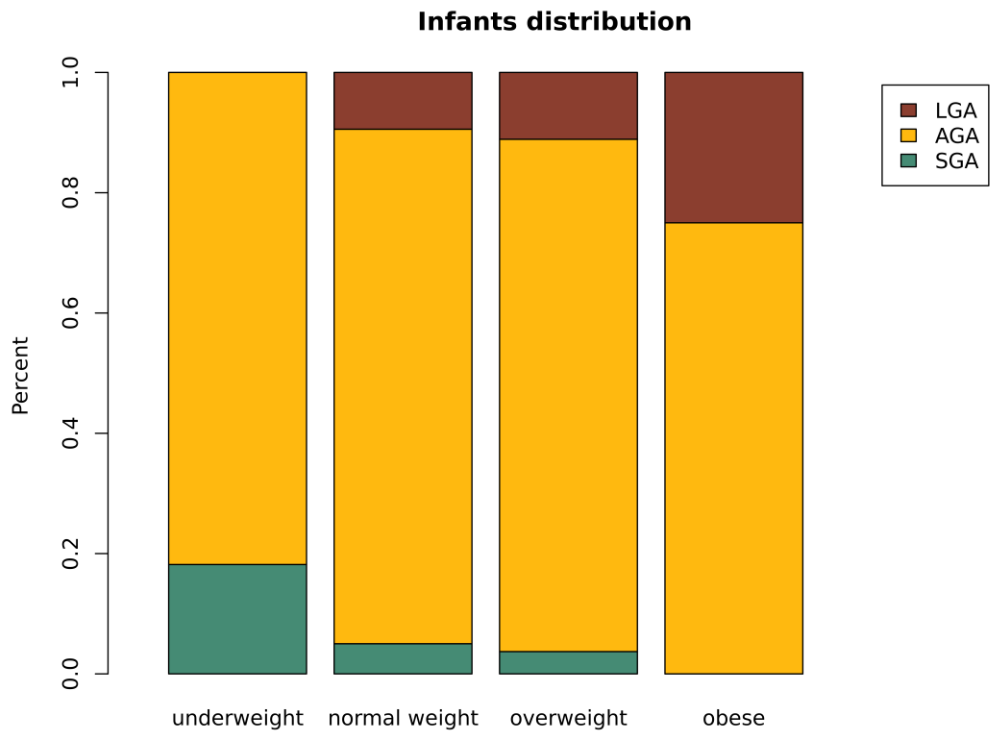

B

	Underweight n=55	Normal Weight n=341	Overweight n=56	Obesity n= 12	BMI Not Available n=39	Total n=503
**SGA**	10 (18%)	17 (5%)	2 (3.6%)	0 (0%)	1 (2.6%)	30 (6%)
**AGA**	45 (82%)	290 (85%)	46 (82.1%)	9 (75%)	27 (69.2%)	417 (83%)
**LGA**	0 (0%)	32 (9.5%)	6 (10.7%)	3 (25%)	5 (12.8%)	46 (9%)
**Birthweigth Not Available**	0 (0%)	2 (0.5%)	2 (3.6%)	0 (0%)	6 (15.4%)	10 (2%)

Figure S2: Infants birthweight distribution in mothers divided by BMI. Infants distribution expressed in percentage (A) and in counts (B) in women categorized as underweight, normal weight, overweight and obese based on the pre-pregnancy BMI.

Abbreviations: BMI, body mass index; SGA, small for gestational age; AGA, appropriate for gestational age; LGA, large for gestational age.

A

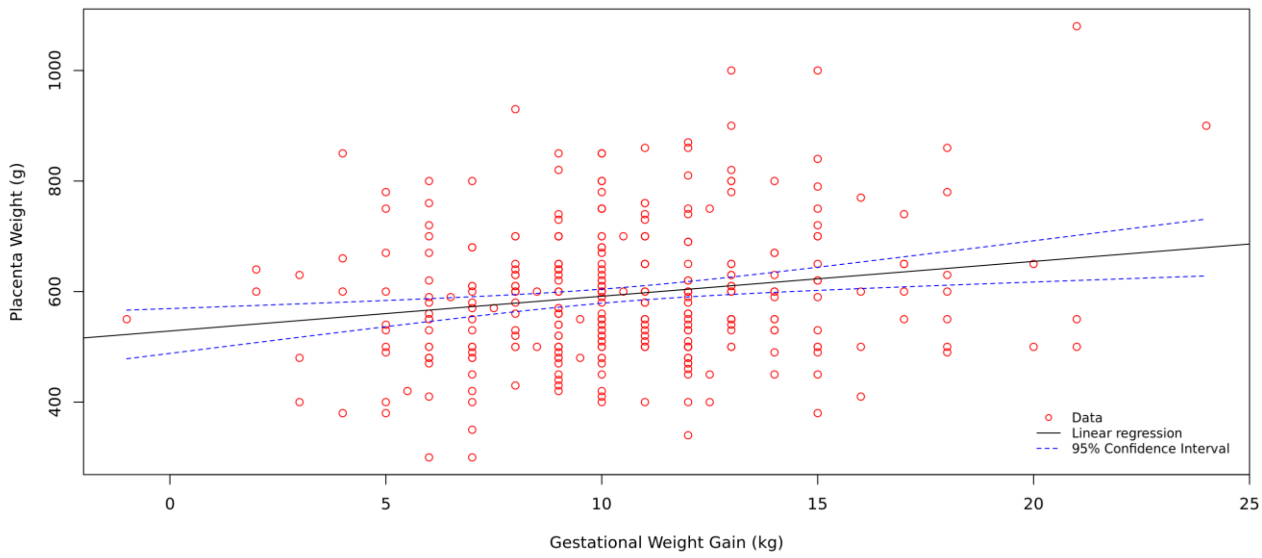

B

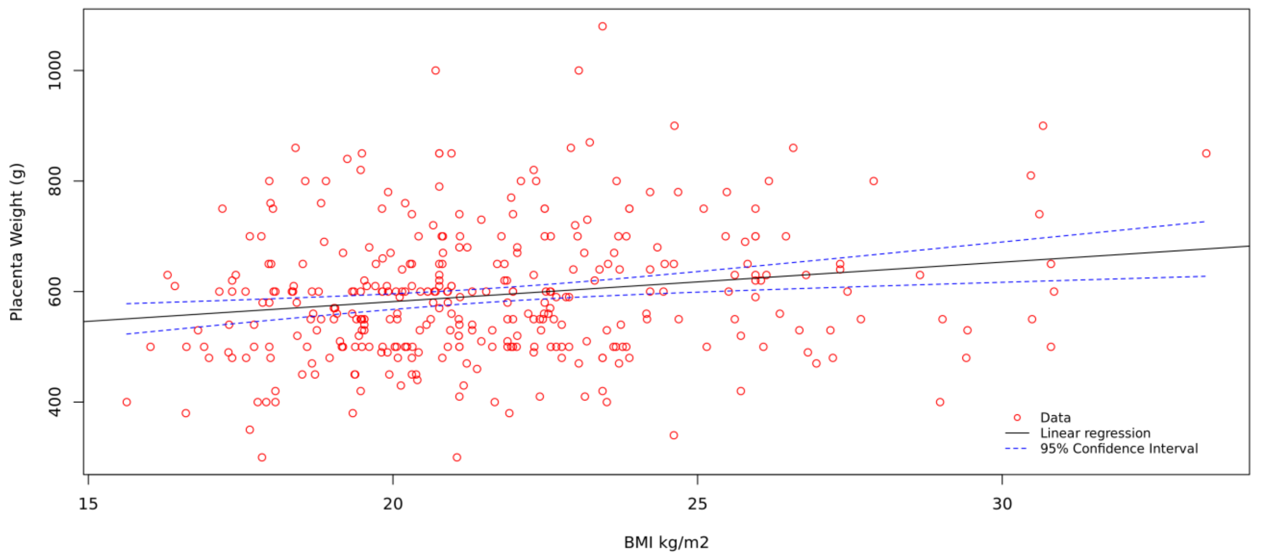

Figure S3: Placental weight and maternal GWG/BMI relationships. Relationships between placenta weight (y-axis) and gestational weight gain (A) and pre-pregnancy BMI (B) obtained from our cohort of 503 women.

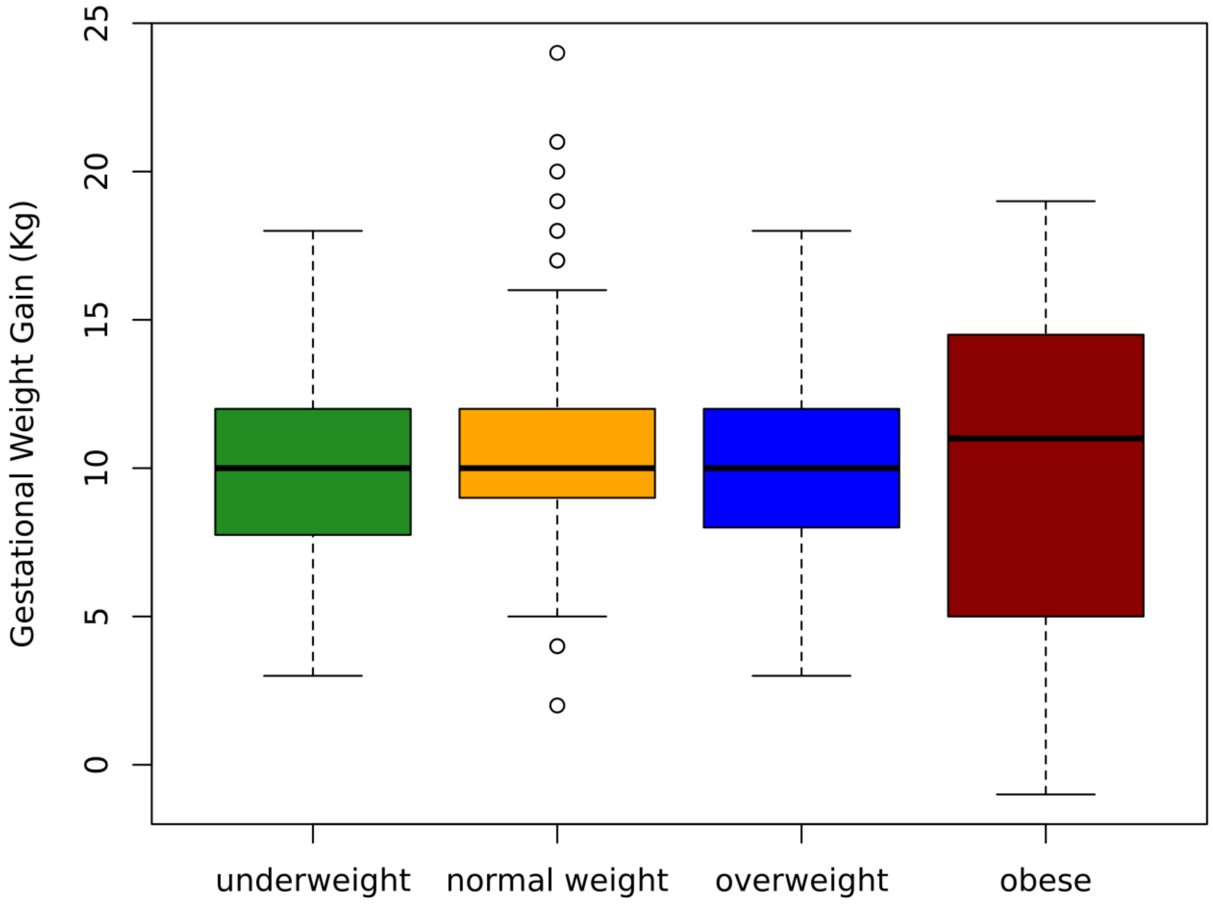

Figure S4: Gestational weight gain distributions in mothers divided by BMI.

Gestational weight gain boxplot distribution in women stratified in underweight (BMI<18.5 kg/m^2), normal weight (18.5≤BMI<25 kg/m^2), overweight (25≤BMI<30 kg/m^2) and obese (BMI≥30 kg/m^2).

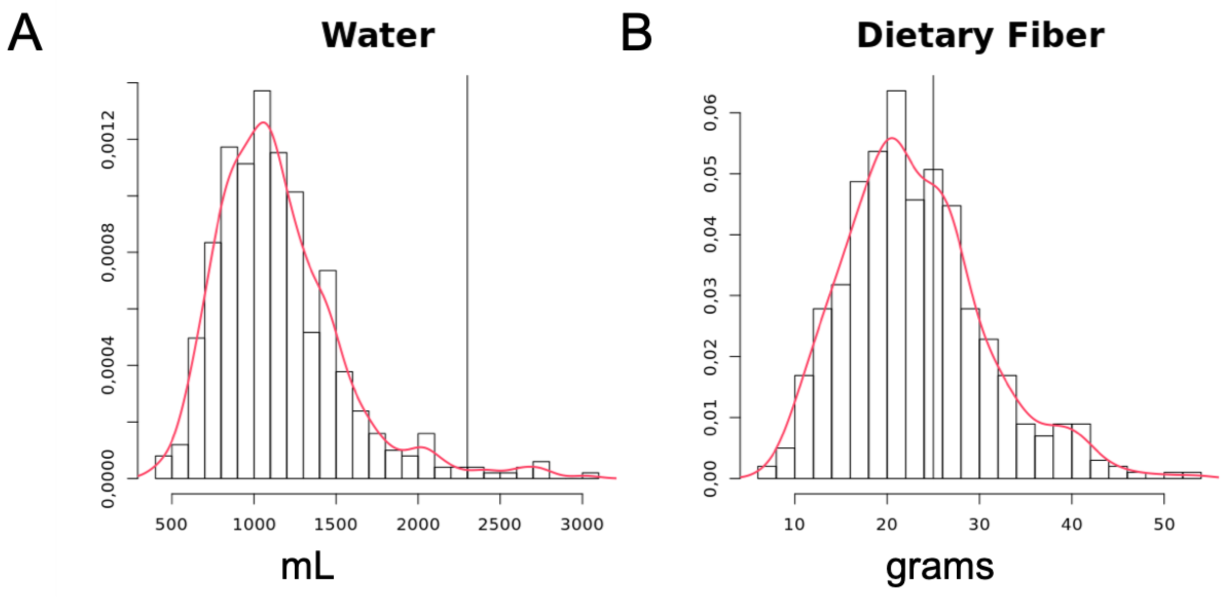

Figure S5: Distribution of daily water (A) and fiber (B) intake in 503 women. The black lines represent the EFSA recommended value.

Table S1. Nutrition and maternal/neonatal anthropometric parameters analysis. Maternal parameters and neonatal birthweight in women following EFSA recommendations for all macronutrients (n = 151/503) and those that fell outside EFSA guidelines for fats, proteins and carbohydrates (n = 11/503).

	**Within EFSA ranges, n = 151**		**Out EFSA ranges, n = 11**	
	Mean ± SD	min-max	Mean ± SD	min-max
**Pre-gestational BMI (kg/m^2)**	22.27 ± 3.7	15.63 – 47.11	21.20 ± 1.4	19.72 – 24.4
**Gestational weight gain (kg)**	10.28 ± 3.9	-1 – 21	10.7 ± 4.5	6.5 – 21
**Placental weight (g)**	577.9 ± 104.9	300.0 – 930	578.3 ± 68.8	500 – 680
**Birthweight (g)**	3355 ± 426.4	2045 – 4490	3053 ± 428.8	2380 – 3920
